# Integration of Transcriptomics, Proteomics, and MicroRNA Analyses Reveals Novel MicroRNA Regulation of Targets in the Mammalian Inner Ear

**DOI:** 10.1371/journal.pone.0018195

**Published:** 2011-04-05

**Authors:** Tal Elkan-Miller, Igor Ulitsky, Ronna Hertzano, Anya Rudnicki, Amiel A. Dror, Danielle R. Lenz, Ran Elkon, Martin Irmler, Johannes Beckers, Ron Shamir, Karen B. Avraham

**Affiliations:** 1 Department of Human Molecular Genetics and Biochemistry, Sackler Faculty of Medicine, Tel Aviv University, Tel Aviv, Israel; 2 Blavatnik School of Computer Science, Tel Aviv University, Tel Aviv, Israel; 3 Helmholtz Centre Munich, German Research Center for Environmental Health (GmbH), Institute of Experimental Genetics, Neuherberg/Munich, Germany; 4 Technical University Munich, Center of Life and Food Sciences Weihenstephan, Freising, Germany; Telethon Institute of Genetics and Medicine, Italy

## Abstract

We have employed a novel approach for the identification of functionally important microRNA (miRNA)-target interactions, integrating miRNA, transcriptome and proteome profiles and advanced *in silico* analysis using the FAME algorithm. Since miRNAs play a crucial role in the inner ear, demonstrated by the discovery of mutations in a miRNA leading to human and mouse deafness, we applied this approach to microdissected auditory and vestibular sensory epithelia. We detected the expression of 157 miRNAs in the inner ear sensory epithelia, with 53 miRNAs differentially expressed between the cochlea and vestibule. Functionally important miRNAs were determined by searching for enriched or depleted targets in the transcript and protein datasets with an expression consistent with the dogma of miRNA regulation. Importantly, quite a few of the targets were detected only in the protein datasets, attributable to regulation by translational suppression. We identified and experimentally validated the regulation of PSIP1-P75, a transcriptional co-activator previously unknown in the inner ear, by miR-135b, in vestibular hair cells. Our findings suggest that miR-135b serves as a cellular effector, involved in regulating some of the differences between the cochlear and vestibular hair cells.

## Introduction

MicroRNAs (miRNAs) are small (17–24 nucleotide-long) non-coding RNAs processed from the transcripts of endogenous genes that function through the RNA interference (RNAi) pathway [1]. Specifically, by binding to sequences in the 3′ untranslated region (UTR) of genes, a miRNA can inhibit target mRNAs. Inhibition occurs either by translational suppression and mRNA destabilization of mRNAs with imperfect complementary sequences, common in mammals, or by cleavage of mRNAs with a perfect match to their sequence, common in plants [2], [3]. In the former, it is believed that conserved pairing to the 5′ region of the miRNA centers on nucleotides 2–7, named the "seed", is important for miRNA target recognition [2]. To date, approximately 200 broad evolutionarily conserved miRNA families and hundreds of additional poorly conserved miRNAs have been identified in mammals [4]. It has been estimated that approximately two thirds of all human protein-coding genes are conserved targets of miRNAs [5]; hence, miRNAs provide a widespread mechanism for posttranscriptional control of gene expression. miRNAs have been implicated in multiple biological processes, including development and differentiation, proliferation, oncogenesis, inflammation, hematopoiesis, and angiogenesis [6]–[10]. Recently, a mutation in miR-96 was found to underlie hereditary hearing loss in humans [11] and mice [12]. To date, this is the only reported example of a miRNA mutation causing a Mendelian disease.

The classical approach to understanding biological roles of miRNAs has been to identify their targets and study their function in the relevant system. However, methods for predicting miRNA targets have proved to be a major barrier in the field, mainly due to the incomplete understanding of miRNA target gene binding interaction. While computational target prediction algorithms provide large lists of proposed miRNA targets, a relatively limited number have been validated. To improve the likelihood of identifying biologically relevant targets, studies often utilize microarray analysis to determine the expression profiles of miRNAs and their predicted target mRNAs (e.g. [8], [13], [14]). Although recent studies demonstrate that repression of proteins is frequently mirrored by decreased transcript levels of miRNA targets [15]–[17], examples where translational repression is the major component of silencing have been identified as well [17]–[19]. Therefore, studying both the mRNA and protein levels provides the most informative view of miRNA regulation and their functional roles in particular tissues or organs.

The mammalian inner ear is composed of the auditory system (cochlea) and the balance system (vestibule). The sensory organs of these systems are specialized epithelia comprised of hair cells and supporting cells. While the cochlea consists of a single sensory organ the vestibule consists of five sensory patches, three at the end of the semicircular canals that sense rotational movement, and the saccule and utricle that sense linear acceleration. Sound, movement and acceleration cause deflection of hair cell apical projections, named stereocilia, located at the luminal surface of the epithelium. This results in an influx of positively charged ions into the cells, creating a graded receptor potential that causes release of a neurotransmitter and stimulates an action potential in the postsynaptic neurons to propagate the signal to the central nervous system [20]. Thus the peripheral auditory and vestibular systems have multiple similarities, with some striking differences in the composition of accessory structures, support cells and hair cell fine structure [21]. Therefore, comparative analysis of the miRNAs expressed in these two systems is likely to identify tissue-specific key regulators of post-transcriptional control of gene expression.

In this study, we identify functionally important miRNA-target pairs in the mammalian inner ear through an *in silico* prediction model that integrates miRNA, mRNA and protein expression. Our approach addresses specific characteristics of miRNA regulation including the number of miRNAs regulating each target, the number of target sites within the target gene 3′ UTR, the 3′ UTR length and the biological context of the regulation. We examined the differential expression landscapes of miRNAs, mRNAs, and proteins in the mouse postnatal day 2 (P2) inner ear cochlear and vestibular sensory epithelia, with its underlying mesenchyme. Thus, we present a comprehensive expression profile of the sensory organs of the inner ear. We found significant enrichment and depletion of targets for 13 and 20 miRNA families, respectively, in the differentially expressed mRNA and protein sets. In nine of the interactions, the miRNA was differentially expressed between the two tissues. Notably, for five of these interactions, the targets were identified only in the protein expression sets. Six miRNA families appear to be functionally important in the inner ear, as demonstrated by the enrichment or depletion of their predicted targets and correlated change in tissue-specific expression. For two miRNAs, miR-135b and miR-205, we localized their cell specific expression in the inner ear using *in situ* hybridization. Furthermore, we demonstrated the translational regulation of PC4 and SFRS1 interacting protein 1 (PSIP1), a transcriptional coactivator previously unknown to function in the inner ear, by miR-135b. Our approach represents a generalizable strategy that can be extended to functional studies of miRNAs in organs or tissues of interest.

## Results

### Identification of miRNAs expressed in the cochlear and vestibular sensory organs

To specifically evaluate miRNA expression profiles from mouse cochlear and vestibular sensory epithelia, the mercury LNA™ miRNA array platform was utilized. This array platform contains quadruplicate probes for 568 miRNAs; of these, 341 are known in mouse and 447 are known in human, with an overlap of 220 miRNAs between the two species.

After filtering out signals of low intensity, 157 miRNAs were detected in one or both tissues (see Methods). Of these, 138 miRNAs were found to be expressed in the cochlea, 146 miRNAs in the vestibule, and 127 in both organ systems (Table S1). Importantly, well established tissue-specific miRNAs, which are not expected to be expressed in the inner ear, such as mir-1/206, mir-155, mir-122 and mir-375, were not detected in either organ by our microarray analysis. Moreover, well characterized inner ear miRNAs, such as the three members of the miR-183/96/182 cluster were detected in both tissues.

**Figure 1:**
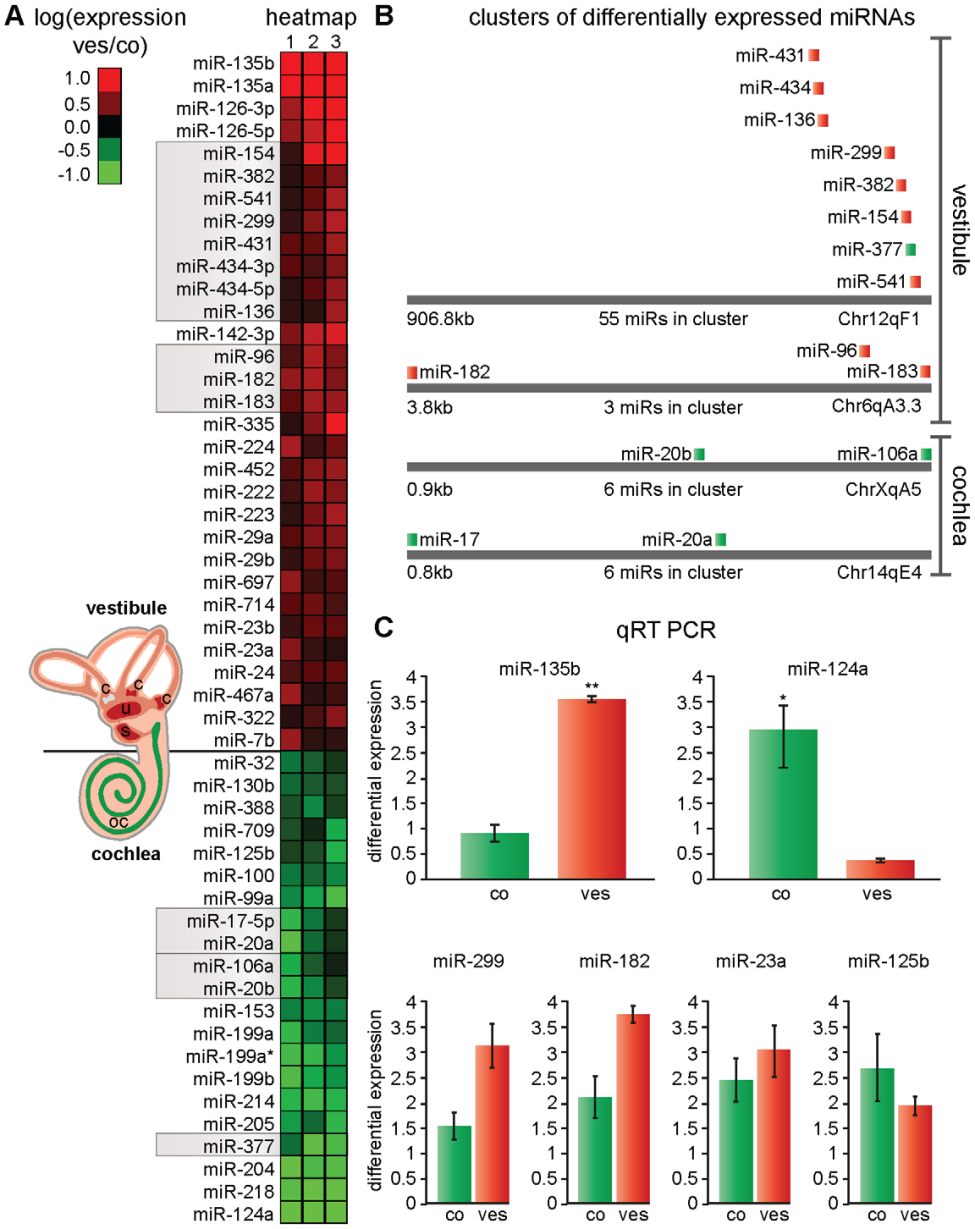
Differential miRNA expression profile in the cochlear and vestibular sensory epithelia. (**A**) Heat map representation of the 52 differentially expressed miRNAs between the P2 cochlear and vestibular sensory epithelia (fold change of at least 1.25 and *P*<0.05). The log_2_ of the ratio between the expression in the vestibular epithelial and the cochlea is shown. Results were averaged for three independent experiments, designated by three columns, each with two technical replicates. miRNAs shown in figure 1B are labeled by a grey box. (**B**) miRNA clusters differentially expressed in the studied tissues. miRNAs up-regulated in vestibule are marked in red and those up-regulated in cochlea are marked in green. Other miRNAs in the same clusters are not shown. miR-377 is the only member of the large miRNA cluster on chromosome 12qF1 significantly up-regulated in the cochlea. (**C**) qRT-PCR analysis of selected miRNAs. miR-135b and miR-124a, the miRNAs with the highest differential expression in the array, exhibited significant differential expression between the cochlear and vestibular sensory epithelia in the validation (4-fold and 8-fold, respectively). n = 3; (*) *P*<0.05, (**) *P*<0.005 versus the other tissue. miR-299, miR-182, miR-23a and miR-125b are representative of varying degrees of differential expression in the array. All PCR results were consistent with the array results. n = 3. Abbreviations: co-cochlea; ves-vestibule; oc-organ of Corti; s-saccule; u-utricle; c-cristea.

We identified 52 miRNAs as being differentially expressed between cochlear and vestibular sensory epithelia with a fold change of at least 1.25 and *P*<0.05 (n = 3, see Methods). Of these, 31 were up-regulated in the vestibule and 21 in the cochlea (Figure 1A). Many of these differentially expressed miRNA genes are clustered together in the mouse genome (Figure 1B). These include the mir-183/96/182 cluster that had a higher expression in the vestibule (fold change of 1.4–1.5) as well as two members of each of the mir-17/92 and mir-106a/363 clusters, which had a higher expression in the cochlea. Interestingly, we found that eight of the miRNAs preferentially expressed in the vestibule were located in the large miRNA cluster on chromosome 12qF1, which contains 55 miRNAs mostly of unknown function [22]. Only one miRNA from this cluster had a higher expression in the cochlea (miR-377).

The differentially expressed miRNAs include four pairs of mature miRNAs derived from the same pre-miRNA hairpin (miR-467a and miR-467a*, miR-434-5p and miR-434-3p, miR-199a and miR-199a*, miR-126-3p and miR-126-5p). As expected, miRNAs derived from the same hairpin were expressed at a similar level in the same tissue.

The miRNAs with the greatest differential expression were miR-135b (expressed 2.5 times higher in the vestibule) and miR-124a (expressed 4 times higher in the cochlea). Their expression was confirmed (*P<*0.05) using quantitative real-time RT-PCR (qRT-PCR). miR-135b was up-regulated 4-fold in the vestibule and miR-124a was up-regulated 8-fold in the cochlea (Figure 1C). Four additional miRNAs, miR-299, miR-182, miR-23a and miR-125b, representing varying degrees of differential expression were validated using qRT-PCR. In all cases the PCR analysis was consistent with results of the microarray analysis (Figure 1C). These results, together with the concordant expression changes identified in miRNAs located in the same genomic cluster, belonging to the same sequence similarity group, and originating from the same hairpin, supports the validity of the above mentioned miRNA array analysis.

### mRNA and protein profiling in the cochlear and vestibular sensory organs

As miRNAs target mRNA stability and translation, we utilized Affymetrix GeneChip® MOE 430 2.0 arrays and mass spectrometry to identify transcript expression and protein levels, respectively in cochlear and vestibular sensory epithelia from P2 mice. Although in some cases miRNAs were shown to repress expression of their targets by 50% or more, recent studies, that identified miRNA targets on a genomic scale, demonstrated that individual miRNAs commonly repressed targets to only a modest degree in the range of 20%–50% [15]. As one of the major goals of our study is to correlate between miRNA and target transcript expression, we therefore used a cutoff of at least 30% difference in our expression analysis and a FDR of 10% using the Benjamini-Hochberg correction for multiple testing. We identified 1,365 genes with greater expression in the vestibular system and 488 with greater expression in the cochlea (FDR<0.1 and fold-change of at least 1.3, Table S2).

In order to functionally characterize transcripts that are differentially expressed between the two tissues, we searched for enrichment of Gene Ontology (GO) ‘biological process’ annotations (see Methods). Table S3 lists the GO biological process annotations enriched at FDR<0.05 for each gene set. Both the cochlear and vestibular gene sets were significantly enriched for many developmental, morphogenesis and differentiation-related GO terms.

In addition to analyzing transcript expression, we completed a comprehensive protein profile of the cochlear and vestibular sensory epithelia. To this end, we implemented a relative quantitative proteomics approach using mass spectrometric analysis of isobaric stable isotope labeled peptides (iTRAQ). We identified a total of 456 proteins in one or both tissues. Applying a threshold of at least 1.3 fold change, 63 proteins were found to be more abundant in the vestibular sensory epithelia, while 48 proteins were found to be more abundant in the cochlear sensory epithelia (Table S4).

To ascertain the relationship between the transcript expression and protein abundance, we compared the differential expression ratios observed in the two datasets. For the 424 genes for which both transcript and protein levels were measured, the correlation between the vestibule to cochlea ratios in the transcript and protein datasets was moderate but significant (Spearman correlation 0.2, *P* = 2.45*10^−5^, Pearson correlation 0.32) (Figure S1A). To detect the global structure of the data, the expression of the genes measured at both the transcript and protein levels was subjected to hierarchical clustering (Figure S1B). This analysis shows clear division of the profiles into branches according to tissue of origin, as protein and mRNA samples from the same tissue were consistently clustered together.

The expression of fourteen genes was found to be enriched in the vestibule by more than 30%, both on the transcript (FDR<0.1) and the protein level, and four genes were found to be significantly higher in the cochlea both on the transcript and the protein level. In both cases, the overlap was highly statistically significant (*P* = 4.58·10−6 and *P* = 0.0012, respectively). Conversely, we identified two instances where transcripts were higher in the cochlea, while the corresponding protein had a higher abundance in the vestibule (*P* = 0.83) (Figure S1C). We did not identify any examples of higher mRNA expression in the vestibule coupled with higher protein level in cochlea.

### Identification of functionally relevant miRNA targets by integration of mRNA and protein expression with *in silico* target prediction

**Figure 2:**
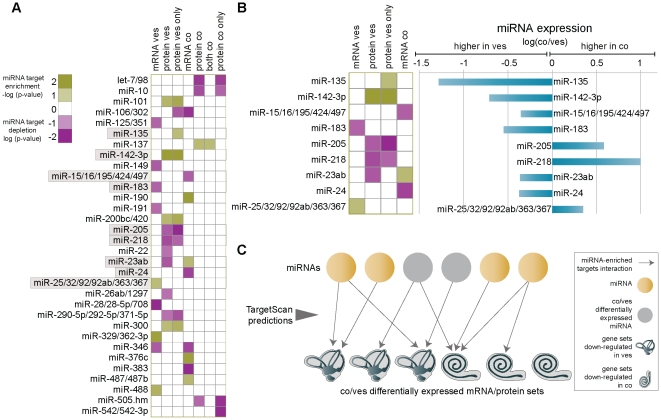
miRNA target identification by integration of mRNA and protein expression with *in silico* target prediction. (**A**) Enrichment and depletion of miRNA targets in co-expressed clusters of mRNAs, proteins, both or proteins only down-regulated in either the cochlea or vestibule. Green squares indicate over-representation of miRNA targets in a cluster and purple squares indicate under-representation. Only miRNA-target cluster pairs with *P*<0.05 are shown. miRNAs shown in figure 2B are labeled by a grey box. (**B**) Differentially expressed miRNAs with significant target enrichments and depletions. (*Left*) Enrichment and depletion of miRNA targets in the co-expressed clusters for miRNAs that showed differential expression based on the miRNA array. (*Right*) The relative expression of the miRNAs on the left represented by the log of the ratio between the expressions in the cochlea as compared to the vestibule. (**C**) An outline of our miRNA target identification approach. Circles represent miRNAs and cochlea/vestibule cartoons represent mRNA/proteins sets that are differentially expressed between the two tissues. Arrows connect miRNA with mRNA/protein sets that are significantly enriched with its predicted targets based on TargetScan predictions. Only miRNAs that have enriched predicted targets in the differentially expressed gene sets are presented. Cases where a miRNA is differentially expressed between the two tissues (shown in B) are marked by a grey circle, while other cases are marked by a brown circle. Abbreviations: co-cochlea; ves-vestibule.

To delineate miRNA targets in the cochlear and vestibular sensory epithelia, we performed a target analysis by combining *in silico* analysis and experimental results. We applied the FAME (Functional Assignment of MiRNA via Enrichment) algorithm [23], a recently developed miRNA functional analysis tool, on our transcript and protein datasets. FAME uses a permutation-based statistical test to detect significant over- or under-representation of miRNA targets in a designated set of genes, utilizing TargetScan 5.0 miRNA target predictions [5]. Unlike standard statistical tests, it utilizes context scores for miRNA-target pairs, and accounts for the number of miRNAs regulating each target and for the number of target sites in the target gene 3**′** UTR [23]. Using the FAME algorithm, we analyzed enrichment and depletion of miRNA targets in the sets of genes down-regulated in one tissue compared to the other at the mRNA level, the protein level or both (see Figure 2C for a description). In addition, as some evidence shows that miRNAs can regulate their targets solely at the protein level, we independently tested a set of genes that were differentially expressed only at the protein level but not at the mRNA level. Overall, these eight sets of genes were tested for enrichment or depletion of target sites for 199 miRNA families that both had target predictions and were represented on our miRNA microarray (Table S5). Based on previous findings [24], conserved predicted targets were used in enrichment tests, whereas both conserved and nonconserved predicted targets were used when testing for depletion.

We found significant enrichment (*P*<0.05) for targets of 13 miRNA families in six differentially expressed mRNA and/or protein sets and depletion of targets of 20 miRNA families in six differentially expressed sets (Figure 2A). Notably, many of the significant enrichments were found only in the proteomics-based sets and not in the mRNA-based sets. Nine of the 33 miRNA families with significant enrichment or depletion in at least one set were differentially expressed between the two tissues in our miRNA microarray data. For these families we found five significant enrichments and eight significant depletions of their targets (Figure 2B). Out of these, nine were in the expected direction. For example, the targets of the mir-135 family, a family that was up-regulated in the vestibule, were enriched in a set of proteins down-regulated in this tissue; and the targets of mir-205, a miRNA that exhibited a higher expression in the cochlea, were depleted in a set of proteins also up-regulated in the cochlea. We noted that targets of mir-135 were marginally enriched in the set of all proteins up-regulated in the cochlea (*P* = 0.075), and a statistically significant enrichment was found only when considering genes up-regulated in the cochlea only on the protein level (i.e., genes without significant mRNA changes between the two tissues, *P* = 0.047). Thus, for six miRNA families – mir-135, mir-205, mir-142-3p, mir-15/16, mir-218 and mir-24 - we obtained evidence for their functional relevance in the inner ear on two levels: (a) the miRNAs were differentially expressed between the two tissues; and (b) their predicted targets were differentially expressed in a manner consistent with the currently accepted model of miRNA regulation.

### Spatial expression of selected miRNAs

**Figure 3:**
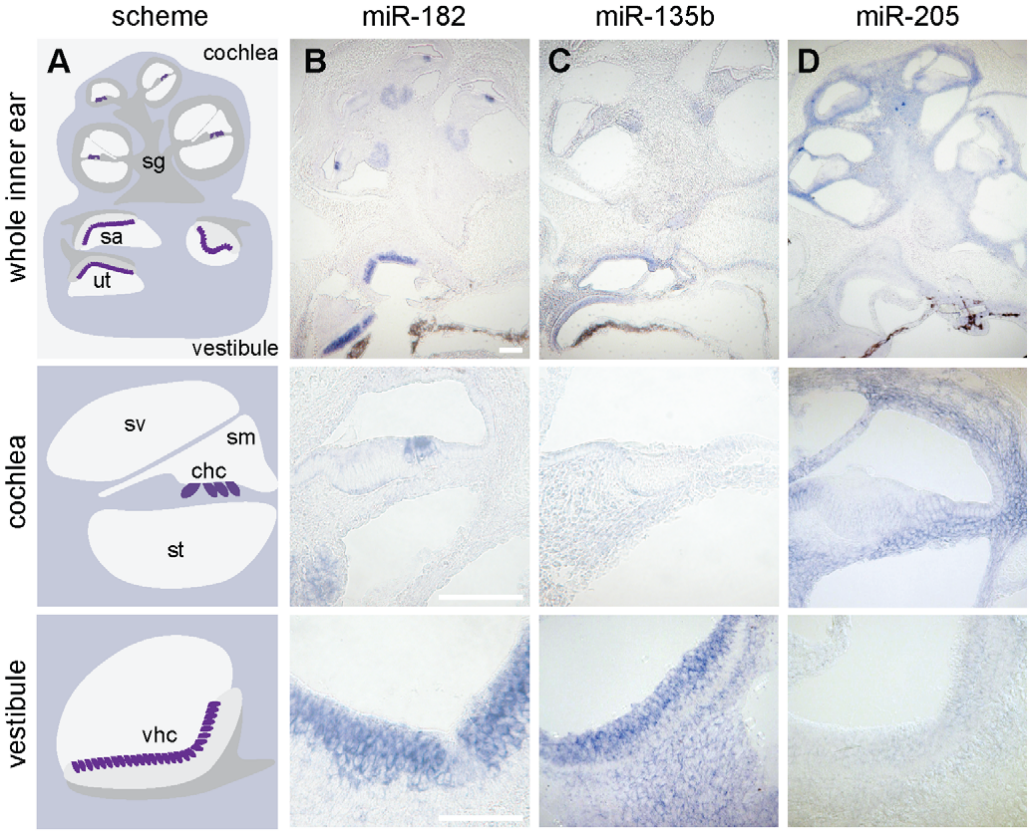
Distinct spatial expression patterns of miR-135b and miR-205 in the newborn mouse inner ear. (**A**) Schematic illustrations of a P0 whole inner ear, cochlea and vestibule: utricle (ut), saccule (sa), spiral ganglia (sg), scala media (sm), scala vestibule (sv), scala tympani (st). In purple, cochlear hair cells (chc) and vestibular hair cells (vhc). (**B-D**) Specific expression patterns were demonstrated for the miRNAs by ISH in whole mount inner ears, followed by cryosectioning. For each miRNA images of the whole inner ear (top) and magnified cochlea (middle) and vestibule (utricle or saccule, bottom) are shown separately. (**B**) A probe for miR-182 was used as a positive control and confirmed probe penetration and staining throughout the inner ear. miR-182 is expressed in the inner ear hair cells and spiral ganglia at this age [31]. Expression patterns for miR-135b (**C**) and miR-205 (**D**) were consistent with the miRNA array analysis. Scale bars: 100 µm.

For further study, we selected miR-135b and miR-205, for which miRNA target enrichment or depletion, respectively, were detected only at the protein expression level and therefore would not have been identified by analyzing transcript data alone. The spatial expression pattern of miR-135b and miR-205 in the inner ear of P0 mice was determined using *in situ* hybridization (ISH; Figure 3), and suggested differences in miRNA function across the cochlea and vestibular organs. Consistent with the miRNA microarray results, miR-135b exhibited specific expression in vestibular organs hair cells. In addition, miR-135b was detected in the neurons of the vestibular and spiral ganglia. As expected from the miRNA microarray data, miR-205 expression was mainly limited to the cochlea. Almost all cochlear cells, including those of the modiolus, were found to express miR-205. Some of the cells in the auditory apparatus did not show miR-205 expression, including many of the cells facing the scala media. We found miR-205 to be expressed in cells of the spiral ligament, part of the Reissner's membrane, basilar membrane and apical surface of the spiral limbus.

### PSIP1 protein expression is up-regulated in the cochlea as compared to the vestibule

**Figure 4:**
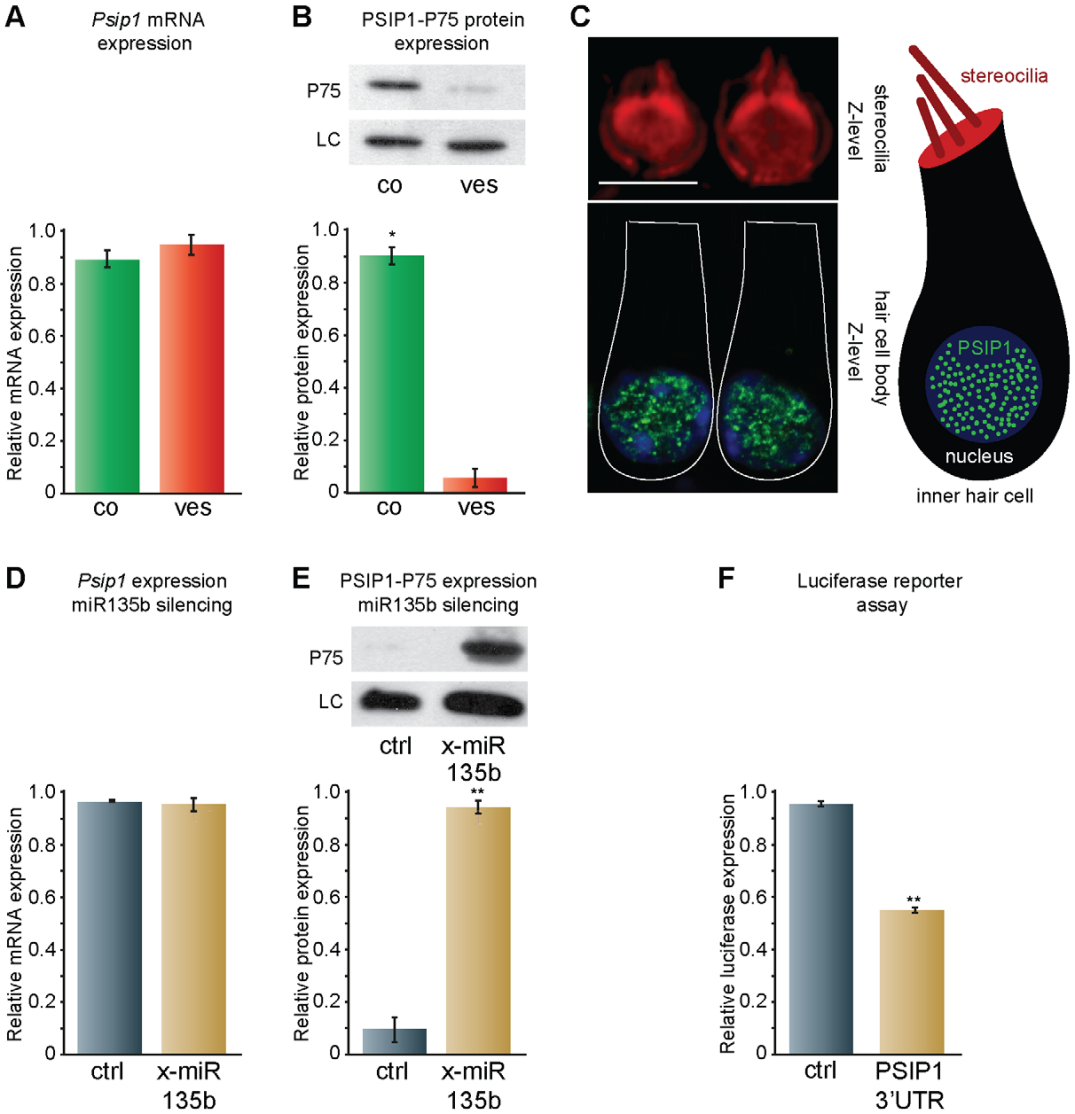
miR-135b regulates PSIP1-P75 expression by inhibition of translation. (**A**) qRT-PCR showing the relative *Psip1* transcript expression in the cochlear and vestibular sensory epithelia (n = 3, *P* = 0.34)**.** (**B**) PSIP1-P75 protein differential expression as measured by western blotting. (*Top*) A representative blot comparing PSIP1-P75 expression in the cochlear and vestibular sensory epithelia. HSC70 loading control is shown for both samples. (*Bottom*) Relative PSIP-P75 expression as measured using the Image J software and following averaging of three independent experiments; (*) *P*<0.05 versus the vestibule. (**C**) Immunolabeling of PSIP1-P75 in P2 cochlear inner hair cells (green). Phalloidin stains the stereocilia (red) and DAPI the nucleus (blue). PSIP1-P75 is localized specifically to the nucleus. Scale bar: 5 µm. A schematic representation of an inner hair cell is shown to the right. (**D, E**) An RNAi system was used to measure the ability of miR-135b to reduce PSIP1-P75 protein expression. shRNA targeting miR-135b (X-miR-135b, oligoengine) or an anti-miR negative control were transfected to Cal51 cells that naturally express miR-135b. After selection, miR-135b and *Psip1* mRNA levels were measured using qRT-PCR and the protein level of the P75 isoform was measured using semi-quantitative western blot analysis. (**D**) qRT-PCR showing the relative *Psip1* transcript expression (n = 3, *P* = 0.66). (**E**) (*Top*) A representative blot showing the relative PSIP1-P75 protein expression. HSC70 loading control is shown for both samples. (*Bottom*) Relative PSIP1-P75 protein expression as measured using the image J software and following averaging of three independent experiments. (**F**) Dual luciferase reporter assay showing the effect of miR-135b on *Psip1-P75*-3′UTR and *Psip1-P75*-3′UTR-M in MCF-7 cells. Relative luciferase expression following averaging of three independent experiments, each conducted in triplicates. (**) *P<<*0.005.

We chose to focus on miR-135b due to its intriguing cell specific expression pattern and its high differential expression between the vestibule and cochlea. Furthermore, our data predict that miR-135b regulates three targets in the vestibule, PSIP1-P75 (also called LEDGF) and PC4, two interacting transcriptional coactivators [25], [26] and ARCN1, a subunit of the coat protein I (COPI) complex required for intracellular trafficking [27]. Each of the targets contains a single sequence complementary to the miR-135b seed within their 3′ UTR. The P52 isoform of PSIP1 does not have a target site within its 3′UTR and is therefore not a potential target of miR-135b. Significantly, this is the first time these proteins have been identified in the inner ear. qRT-PCR analysis confirmed the presence of *Psip1* transcript in the sensory epithelia of both the cochlea and the vestibule, and quantification revealed a similar expression level in the two tissues (Figure 4A; n = 3, *P* = 0.34; Student's *t*-test). Semi-quantitative western blot showed on average 9-fold decrease (n = 3, *P*<0.05; Student's *t*-test) in PSIP1-P75 protein expression in the vestibular as compared to the cochlear sensory epithelia (Figure 4B). Together, these data demonstrate that while *Psip1* is transcribed equally in the cochlear and vestibular sensory epithelia, the translation to the P75 isoform of this protein is inhibited in the vestibular epithelia.

The presence of PSIP1-P75 isoform in the inner ear was further confirmed using immunohistochemistry. Its expression was distributed heterogeneously in the nucleoplasm of the hair (Figure 4C) and supporting cells both in the cochlea and vestibule, forming a speckled pattern. Such a finding is consistent with previous reports of PSIP1-P75 localization to chromatin in both interphase and mitotic chromosomes [28], [29]. Although PSIP1-P75 displayed expression in both the cochlea and vestibule, the level of expression cannot be compared quantitatively by this technique.

### miR-135b down-regulates PSIP1-P75 protein expression

The functional interaction between miR-135b and PSIP1-P75 was further determined by *in vitro* analysis utilizing an RNAi approach and a luciferase reporter assay. Cal51, breast carcinoma, cells were found to express high levels of miR-135b, and relatively low levels of PSIP1-P75. Cal51 cells were transfected with either a plasmid expressing shRNA targeting miR-135b or an anti-miR negative control. qRT-PCR analysis revealed a reduction in miR-135b expression and similar *Psip1* mRNA expression level in both the cells transfected with the shRNA targeting miR-135b and transfected with the anti-miR negative control (Figure 4D; n = 3, *P* = 0.66; Student's *t*-test). Semi-quantitative western blot analysis exhibited a 10-fold increase in PSIP1-P75 protein abundance in the cells expressing shRNA targeting miR-135b (Figure 4E; as compared to transfection with an anti-miR negative control. n = 3, *P<<*0.005; Student's *t*-test). Subsequently, we subcloned the 3′UTR of *Psip1-P75* downstream of a luciferase gene, creating a Luc-*Psip1-P75*-3′UTR vector. Transient co-expression of this vector with a miR-135b expressing vector revealed that miR-135b reduced luciferase expression levels by approximately 40% (Figure 4E; as compared to a mutated Luc-*Psip1-P75-*3′UTR control. n = 3, *P*<<0.005; Student's *t*-test). These results indicate that miR-135b down-regulates the expression of PSIP1-P75 protein but does not affect the mRNA levels.

## Discussion

We have employed a novel and generalizable method to efficiently identify functional miRNA-target interactions in a neuroepithelial tissue. This approach can be extended to any tissue or organ of interest. Specifically, we investigated the differences between sensory epithelia of the cochlear and vestibular portions of the inner ear, in an attempt to decipher critical elements driving differential gene regulation in each system.

In this study we describe a miRNA profile of the inner ear sensory epithelia. To date, two studies profiled the miRNAs in the whole inner ear and whole cochlea and vestibule [30], [31]. By focusing on a more specific tissue, our work aims to provide depth and understanding of the inner ear organs with a primary sensory role. We report the presence of 157 miRNAs in at least one of the sensory epithelia in the inner ear, with 52 differentially expressed between the cochlea and vestibule. A close analysis of the miRNA differential profile revealed that many of the miRNAs co-expressed in the studied tissues are clustered in the mouse genome. This observation is consistent with the notion that clustered miRNAs are usually expressed together as polycistronic, co-regulated units [32]. The vestibular up-regulated miRNAs include all the members of the miR-183/96/182 cluster previously demonstrated to be specifically expressed in the mammalian inner ear hair cells and ganglia [11], [12], [31]. As the amount of RNA obtained from the dissected tissues is greater in the cochlea, due to its larger size, the differential expression of these miRNAs might be due to the different percentage of hair cell specific RNA in the total RNA from each of the two tissues.

Specific expression profiles of a miRNA in a given tissue may point to the particular role of the miRNA in that tissue. It has been suggested that miRNAs serve as cell effectors among cells of related fates [33]. Furthermore, it is believed that miRNAs play an important role in terminal differentiation and maturation of different cell types within a particular cell lineage, as well as regulating cellular processes in differentiated cells during morphogenesis and homeostasis (reviewed in [34]). The cochlear and vestibular sensory epithelia share many similarities and differences. Specifically, the sensory cells embedded in these tissues function through the same mechanotransduction mechanism and have a similar but not identical morphology. Both cell types have stereocilia projections arranged in bundles but the shape and arrangement of the bundles is different in the two systems [35], [36]. Importantly, cochlear hair cells are unable to regenerate in the mammal, while early vestibular hair cells are able to do so to some extent [37], [38]. We speculate that the miRNAs differentially expressed between the cochlea or vestibule may participate in regulating these tissue identities and maintaining their distinct function.

The present study expands on the known inner ear transcript and protein profiles. We characterized the repertoire of differentially expressed transcripts and proteins in the vestibular system as compared to the cochlea. Several of the genes identified in our analyses were previously studied in the inner ear and a few (e.g., crystallin [39] and cochlin [40]) have been shown to cause deafness. However, many of the genes found to be expressed in the inner ear sensory epithelia, according to the transcript and protein datasets, have not been identified in the inner ear thus far, and their functional role is yet unknown. Notably absent from the proteomic dataset are hair cell-specific proteins. This is likely due to the limitation of the iTRAQ mass-spec method to identify low abundance proteins. The tissues studied contain different cell types, making it difficult to predict the function of genes and proteins within specific cell types. In order to understand their functional relevance, the proteins identified would have to be studied in depth individually.

The correlation between the vestibule to cochlea ratios of the mRNA and the protein levels was relatively low, though significant. This could be due to the limited protein expression data or a relatively high level of post-transcriptional regulation. Similar correlations between mRNA and protein changes were previously observed in analyses of embryonic mouse brain tissues [41], in gastric cancer cells [42] and in the yeast *Saccharomyces cerevisiae* [43].

Currently, miRNA target identification is based primarily on computational target predication algorithms. The vast number of targets predicted by these algorithms raises the problem of choosing which of these are worthy for experimental validation. For example, searching for the potential targets of the 52 differentially expressed miRNAs using the TargetScan algorithm led to the identification of 11,031 putative conserved targets. Therefore, to narrow down the targets list and to detect miRNA-target pairs with a higher likelihood for successful validation, we utilized a strategy that combines *in silico* analysis and experimental techniques.

To analyze enrichment or depletion of miRNA targets we applied the FAME algorithm on our datasets of differentially expressed transcripts and proteins. Genes preferentially co-expressed with a miRNA have evolved to avoid targeting by that miRNA [24]. Thus, depletion of targets is expected for genes that are expressed in the same tissue as the miRNA [24], [44]. We therefore focused on miRNAs and targets with a reciprocal expression and miRNAs and anti-targets (messages selectively avoiding targeting to a miRNA; see [45]) with a similar expression pattern. In some cases, miRNAs and their potential targets were observed to have a similar expression pattern, and not a reciprocal one as expected. Such a phenomenon might be explained by the counter regulation of different posttranscriptional control mechanisms or by miRNA induced translation up-regulation as previously observed for the miRNAs miR-369-3 and let-7 in cell cycle arrest [46]. We note that some of the miRNA targets predicted by the analysis could only be detected using our proteomics data, while others were only identified using the transcriptomics data. Thus by looking at both levels of expression we were able to identify the most thorough list of miRNA-target pairs. It should be pointed out that our power is limited by the detection constraints of the proteomics screen, and thus we expect this list to be only partial.

The most notable miRNA for which we identified translationally repressed targets was miR-135b, the miRNA with the highest differential expression in our dataset. miR-135b is located within the first intron of the *LEM domain containing 1* (*Lemd1*) gene. Interestingly, our Affymetrix microarray analysis showed a high expression of *Lemd1* in the vestibular sensory epithelia. Therefore, it is likely that miR-135b is transcribed as part of *Lemd1*, leading to a similar expression pattern. To better understand miR-135b function in the inner ear, we studied its spatial expression. *In situ* hybridization demonstrated specific expression of miR-135b in vestibular hair cells. No such expression was observed in the cochlea, consistent with our microarray and qRT-PCR results. The distinct expression pattern of miR-135b most probably points to a specific regulation mechanism that exists in the vestibular hair cells but not in the cochlear hair cells. To date, the only miRNAs identified demonstrating inner ear hair cell specificity are part of the miR-183/96/182 family [31]. Unlike miR-135b, these miRNAs are expressed both in the cochlear and vestibular hair cells.

Of the three putative targets of miR-135b, we chose to further validate the interaction with the P75 isoform of PSIP1. PSIP1 is a transcriptional coactivator involved in neuroepithelial differentiation and neurogenesis [47]. In particular, it plays a role in gene regulation in the epithelial cells of the lens and is considered to be involved in cell fate determination [48]. Such functions correspond well to possible involvement in the differentiation and maintenance of the sensory epithelia in the inner ear. It is therefore not surprising that PSIP1 is expressed in the inner ear sensory epithelia, as demonstrated in this study. The *Psip1* gene is alternatively spliced into two different isoforms; *P75*, the larger isoform, and *P52* [26], [49]. Of the two, only *P75* contains a sequence within its 3′ UTR with the potential of being targeted by miR-135b. Using qRT-PCR and semi quantitative western blot analysis, we were able to demonstrate inhibition of PSIP1-P75 protein expression in the vestibular sensory epithelia suggesting intervention by a translational regulation mechanism. *In vitro* analysis further proved an interaction between miR-135b and PSIP1-P75. Interestingly, the efficiency by which miR-135b silences PSIP1-P75, as identified by our *in vitro* analysis, is much higher than previously expected for targets with only a single binding site for a miRNA seed [2]. Due to the limitation in efficient transfection of inner ear organotypic cultures, we could not show the direct interaction *in vivo*. Taken together, our results demonstrate the regulation of PSIP1-P75 by miR-135b in vestibular hair cells. According to our results, miR-135a also has a higher level of expression in the vestibular as compared to the cochlear sensory epithelia. Due to the similarity between miR-135b and miR-135a, we predict that miR-135a also regulates PSIP1-P75 in the vestibular system.

**Figure 5:**
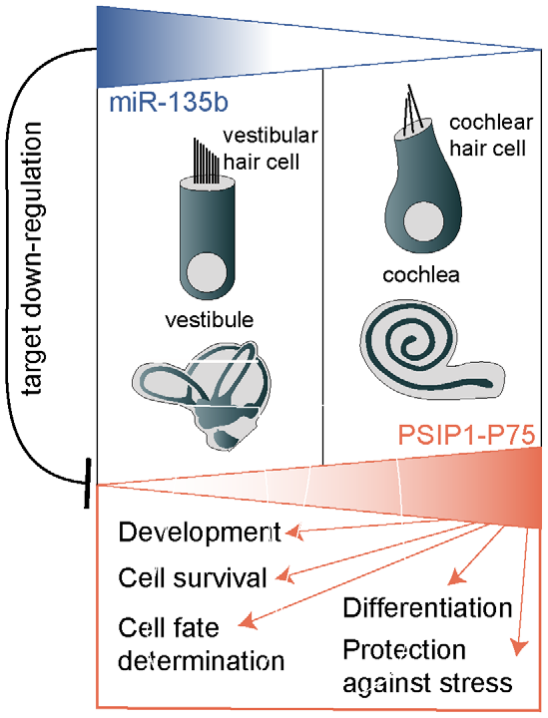
Schematic representation of a possible role of miR-135b in the inner ear. miR-135b (blue) is expressed in the vestibular hair cells, whereas no expression is detected in the cochlear hair cells. miR-135b down-regulates PSIP1-P75 (red) expression in the vestibular hair cells while its expression remains relatively high in the cochlear hair cells. Due to this differential expression, PSIP1-P75 regulates (red arrows) different process in the cochlear hair cells and only to a lesser extent in the vestibular hair cells. These processes include development, differentiation, cell fate determination, cell survival and protection against stress.

The overall effect of miR-135b in the inner ear is summarized in Figure 5. In this scheme, we propose a unique mechanism by which miR-135b down-regulates PSIP1-P75 expression in the vestibular hair cells, whereas it remains relatively high in the cochlea. Thus the effect of PSIP1-P75 transcriptional regulation is more pronounced in the cochlear hair cells, leading to downstream perturbation that possibly influences the cell's identity, differentiation and maintenance. PSIP1-P75 was previously shown to be involved in cell survival [50], protection against stress [51], differentiation [52], cell fate determination [48] and is believed to regulate genes involved in development [53]. We hypothesize that PSIP1-P75 and miR-135b might play a role in regulating these processes in the cochlear hair cells, whereas in vestibular hair cells they are modulated by other miRNA. By this means, miR-135b might serve as a cellular effector, involved in regulating the differences between the cochlear and vestibular hair cells and thus contributes to their distinct cell identities and maintaining their specific functions. It should be pointed out that these processes involve more intricate mechanisms that have yet to be revealed, including the interplay among different miRNAs and proteins.

In this work we found evidence for the functional importance of many previously unknown inner ear sensory epithelia miRNAs. We reason that miRNAs differentially expressed between the cochlear and vestibular sensory epithelia may participate in regulating the cellular identities of these tissues and maintaining their distinct morphology and function. Using our target analysis approach, we were able to identify both miRNA targets affected at the mRNA level and ones only affected at the protein level. Significantly, the identification of a bona-fide miRNA-target pair, miR-135b and PSIP1-P75, predicts a role for this pair in inner ear cell survival, protection against stress, differentiation, cell fate determination and development, and may explain differences in regeneration of vestibular vs. cochlear hair cells.

## Materials and Methods

### Animal handling

All procedures involving animals met the guidelines described in the National Institutes of Health Guide for the Care and Use of Laboratory Animals and have been approved by the Animal Care and Use Committees of Tel Aviv University (M-07-061 and M-08-026).

### Dissection approach

For miRNA, mRNA and protein expression profiling, as well as the qRT-PCR and western blot analysis, cochlea and vestibular sensory epithelia were dissected from P2 wild type C3H mice and collected separately. The vestibular epithelia consisted of the saccule, utricle and the lateral and anterior cristae. Both the cochlear and vestibular sensory epithelia were dissected with their underlying mesenchyme and attached neurons. Altogether three pools of each tissue type were collected consisting of cochlear or vestibular sensory epithelia dissected from 10 to 12 inner ears.

### Microarrays and bioinformatics analysis

For miRNA expression profiling, dissections were conducted as described above. Small RNA-containing total RNA was extracted from samples using the miRVana™ miRNA isolation kit (Ambion). RNA quality was assessed using a nano-range bioanalyzer (Agilent Technologies). miRNA microarrays, produced using an oligonucleotide probe library (miRCURY LNA array ready to spot) purchased from Exiqon, were a kind gift from Dr. Noam Shomron. Five micrograms of sample RNA were directly labeled with either Hy3 or Hy5 using the miRCURY LNA array labeling kit (Exiqon). Hybridization and washing of the microarray slides were performed as recommended by Exiqon. In order to overcome the difficulty of variations in spotted arrays, three biological repeats were performed, each with two technical repeats (dye swaps). Scanning was performed using an Agilent DNA microarray scanner. The SpotReader software (Niles Scientific) was used to generate raw intensity data. The array data was normalized such that the average intensity in each sample was the same. For each experiment color-swap technical replicates were averaged and a single sample *t*-test analysis was performed to extract differentially expressed genes. A miRNA was considered detected if the spot intensity was at least two standard deviations above background in at least two of the samples.

For mRNA profiling, dissections were as detailed above. Total RNA was extracted using a standard Trizol protocol. RNA was DNAseI treated (Qiagen), resuspended and quality assessed using a nano-range bioanalyzer (Agilent Technologies). RNA was then amplified using the Affymetrix two-cycle kit and hybridized to the Affymetrix GeneChip® MOE 430 2.0 arrays as previously described [41], [54], [55]. Three biological experiments were conducted, each with three replicates. Expression levels were computed using the robust multiarray average (RMA) method (implemented in the BioConductor package). To remove systematic biases among the chips, they were normalized using the quantile normalization scheme. To filter out probe sets whose corresponding genes are not expressed in the analyzed samples, Presence flags were computed according to Affymetrix MAS5 method. Probe sets that got no 'Present' calls were filtered out, leaving 29,636 probe sets for subsequent analysis. Multiple testing was accounted for by correcting the p-values using the Benjamini-Hochberg method to control for the false discovery rate (FDR). Hierarchical clustering with average linkage was performed using Expander [56]. GO analysis on differentially expressed genes was performed using a hypergeometric test, and corrected for multiple testing using the Benjamini-Hochberg FDR method.

The gene expression dataset reported in this publication is MIAME compliant and was deposited in the NCBI's Gene Expression Omnibus (GEO) database, NCBI [**57**] (GEO series accession number GSE23081).

### qRT-PCR

For qRT-PCR analysis of miRNAs expression in the inner ear, dissections were conducted as detailed in the dissection approach. For miR-135b regulation analysis in shRNA transfected Cal51 cells, transfected cells were lysed and frozen at -80°C. Small RNA-containing total RNA was extracted from samples using either the miRVana™ miRNA isolation kit (Ambion) or the miRNeasy Mini Kit (Qiagen). Specific miRNA and U6B RNA (endogenous control) were reverse transcribed using the Taqman Reverse Transcription kit (Applied Biosystems) and their expression was measured using the Taqman microRNA qRT-PCR kits and the ABI Prism 7900 PCR machine (Applied Biosystems). Reactions were performed as three independent experiments, each with three replicates. The miRNAs expression was normalized to the expression of U6B.

For gene expression analysis, total RNA was extracted from the separately collected epithelia using either the RNeasy Plus Mini Kit (Qiagen) or the miRNeasy Mini Kit (total RNA protocol, Qiagen). Total RNA was converted to single stranded cDNA using the High Capacity cDNA Reverse Transcription Kit (Applied Biosystems). qRT-PCR was performed using Taqman Gene Expression Assay for *Psip1* and either *Tbp* or *RPLP0* and the 2X Universal PCR Master Mix (Applied Biosystems). Three experiments were performed, each in triplicates.

### Quantitative protein profiling and mass spectrometry

Mass spectrometry was performed at the Smoler Proteomics Center, Technion, Haifa, Israel. For quantitative protein profiling, cochlear and vestibular sensory epithelia were dissected and collected as described in dissection approach. Each pool consisted of either cochlear or vestibular sensory epithelia collected from 50 to 60 inner ears. Proteins were extracted using a standard Trizol protocol. Protein pellets (50 µg each) were resuspended separately in 8 M urea in 100 mM ammonium bicarbonate and were reduced (10 mM DTT) and modified with 40 mM iodoacetamide. The samples were diluted to 2 M urea with water and the proteins were trypsinized with 2 µg bovine trypsin at 37°C overnight (12 to 16 h). The resulted peptides were cleaned on disposable Silica-C18 tip (Harvard) and resuspended in 100 mM Hepes (pH 7.3). The iTRAQ™ Reagent (Applied Biosystems) was brought to room temp and then mixed with ethanol (30∶70). After vortexing and spinning, each one of the reagents was transferred to one sample tube. The tubes were incubated at room temperature for 1 h. The two iTRAQ™ reagent-labeled samples were combined, cleaned on C18 and resuspended in 0.1% formic acid. 60 µg of the combined labeled peptides were separated in an on-line two dimensional chromatography experiment (MuDPiT). First the peptides were loaded on 15 mm of BioX-SCX column (LC Packing) and eluted with 8 salt steps of 0, 40, 100, 150, 200, 300, 500 mM and 1000 mM ammonium acetate in 5% ACN and 0.1% acetic acid, pH 3. The eluted peptides were further resolved by capillary reverse-phase chromatography (75 µ ID, 30 cm fused silica capillaries, J&W self-packed with 3 µl Reprosil-Aqua C18). The peptides were eluted using a 125 min gradient (5% to 40% acetonitrile containing 0.1% formic acid) followed by a wash step of 95% acetonitrile for 15 min. The flow rate was about 0.2 µl/min and the peptides were analyzed using QTOF-Premier mass spectrometer (Waters). Mass spectrometry was performed in a positive mode using repetitively full MS scan followed by collision induces dissociation (CID) of the 3 most dominant ion selected from the first MS scan.

The mass spectrometry data was clustered, analyzed and compared using the Pep-Miner [58] and Sequest software against the mouse part of the nonredundant (nr) database (NCBI). Quantitative analysis was done using an on-house tool comparing the intensity of 114 and 115 ions in each MSMS spectrum. Only proteins with three or more peptides were considered valid for the ensuing analysis. For each peptide, the ratio between the iTRAQ label peak value and the sum of intensities was calculated. The ratio was normalized in relation to one and divided by the median of the ratios. The raw proteomic data is attached as Table S6.

### Western blot analysis

For comparison of PSIP1-P75 protein expression in the inner ear sensory epithelia dissection was conducted as described above. For miR-135b regulation analysis in shRNA transfected Cal51 cells, transfected cells were spun-down and frozen at -80°C. In both cases, proteins were extracted using NP40 supplemented with X100 protease and phosphotase inhibitors (Calbiochem and Sigma respectively) and abundance was measured using a Bradford assay (Sigma). Equal quantities of lysates were separated by 8% SDS polyacrylamide gels, and electrophoretically transferred to polyvinylidene difluoride membranes (Millipore). Proteins were analyzed by western blotting using rabbit anti-PSIP1-P75 antibody (Cell Signaling Technology) and mouse anti-HSC70 antibody (Santa Cruz Biotechnology Inc.). HSC70 loading control was used to normalize the abundance of specific proteins. Specific proteins relative quantities were calculated using the Image J software (NIH) with subsequent averaging of three separate experiments.

### *In situ* hybridization

At least three independent ISH experiments were performed with each probe, and at least 4 inner ears were included in each experiment. Inner ears of C57BL/6J new born mice were fixed in 4% paraformaldehyde (PFA). Whole mount *in situ* hybridization analysis was performed according to the Exiqon protocol with a few modifications [30]. Briefly, the tissues were incubated in hybridization solution with miRCURY LNA™ microRNA detection probes (Exiqon), at 20**–**22°C below the melting temperature of the probe. The LNA™ digoxygenin (DIG) labeled probes were detected by anti-DIG-AP (alkaline phosphatase conjugated) antibody (Roche). NTB/BCIP (Sigma) was added to develop the color reaction. The tissues were then cryosectioned to 10**–**18 µm sections using the LEICA CM3050S cryostat. The sections were mounted and imaged using the Ziess Axiovert200 M microscope.

### miRNA Target prediction and enrichment analysis

miRNA target predictions were obtained from TargetScanMouse 5.0 (http://www.targetscan.org/mmu_50/). All the conserved targets for conserved miRNA families were used for over-representation analysis, and both conserved and nonconserved targets for conserved miRNA families were used for under-representation analysis. FAME algorithm is described in detail in [23]. Briefly, TargetScan predictions are used to construct a weighted bipartite graph in which miRNAs are connected to their predicted targets. The edge weights are based on the context scores assigned by TargetScan to each miRNA target site. For each miRNA and each gene set, the total weight of the edges between the miRNA and the genes in the set is compared to the weight expected based on random perturbations of the bipartite graph, which preserve the number of miRNAs targeting each gene and the number of targets for each miRNA. This comparison is used to derive an empirical p-value for the enrichment of miRNA targets in the gene set.

### Cell culture and transfection

Cells from the Cal51 [59] and MCF-7 (HTB-22™, ATCC), breast carcinoma cell lines, were grown in DMEM supplemented with 10% FCS (Beit Haemek Biological Industries) and antibiotics (Invitrogene). For miR-135b inhibitor treatment, Cal51 cells were transfected using JetPEI reagent (Polyplus transfection™) with a pSUPER-GFP plasmid encoding either shRNAs against mouse miR-135b or negative control shRNA (Oligoengine). Cells were cultured for 48 h before selection with G418 Sulfate (Calbiochem). Transfected Cal51 cells were grown in G418 Sulfate until all non-transfected cells died, approximately a week after selection initiation. Total RNAs and proteins were extracted from transfected and selected Cal51 cells as described above. Three separate experiments were conducted.

### Luciferase reporter assay

Luc-*Psip-P75*-3′UTR was produced by subcloning the 3′UTR of *Psip1-P75* into the pGL3 vector (Promega) downstream of the luciferase gene by PCR with TCT AGAG GGA TTT CAG TGG CAT TAG AA (forward) and TCT AGA AAC TTT AAT TAA AAC AAT TTA CAC (reverse) primers. A control construct, Luc-*Psip1-P75*-M, was created by removing the sequence, complementary to the miR-135b seed, using a AGT GTC AAT GTG TAA ATT GTT TTA A phosphor-primer and a AAC TAG AAT AAT TTT TGT CCA AGT T primer and the QuikChange II Site-Directed Mutagenesis Kit (Agilent).

The luciferase reporter assay was performed as previously described [60]. Briefly, MCF-7 cells were grown in 24-well plates and transfected using JetPEI reagent (Polyplus transfection™) with either 5 ng of Luc-*Psip1-P75*-3′UTR or a mutated control, 5 ng Renilla and 0.5 µg of miR-135b (miRNA expression vector obtained as a gift from Reuven Agami, [61]). Luciferase activity was measured 72 h after transfection using the Dual-Luciferase® Reporter Assay System (Promega). Three experiments were conducted, each in triplicates.

### Immunohistochemistry

Whole mount immunohistochemistry was conducted as previously described [62]. Briefly, inner ears from C3H P2 mice were fixed overnight in 4% PFA (Electron Microscopy Sciences) in Dulbecco's phosphate-buffered saline (D-PBS) and washed in D-PBS after which further fine dissection isolation of the cochlea and vestibular sensory organs was conducted. Following permeabilization and blocking, the tissues were incubated overnight at 4°C with rabbit anti-PSIP1-P75 specific antibody (Cell Signaling) diluted in D-PBS (1∶50). Immunolabeling was visualized with an Alexa 488-conugated donkey anti-rabbit antibody (diluted 1∶500; Invitrogen), together with rhodamine phalloidin (diluted 1∶250; Invitrogen), an actin fluorescent dye. After washes, samples were mounted on glass slides using ProLong gold antifade reagent (Invitrogen). Confocal laser microscopy was carried out with a Leica TCS SP5 laser confocal microscope.

## Supporting Information

Figure S1 Relationship between differential cochlear and vestibular transcript and protein datasets. (A) Scatter plot representation of the protein cochlea to vestibule expression ratios obtained by the iTRAQ labeling and MuDPiT analysis and mRNA cochlea to vestibule expression ratios obtained from the Affymetrix microarray. Both expression ratios are plotted in a logarithmic scale. (B) Cluster dendrogram of the differentially expressed mRNA and protein datasets. (C) Intersection of the Affymetrix microarray (pink) and proteomic results (blue). Left diagram: Genes significantly up-regulated in the cochlea both on the mRNA and the protein levels. Middle diagram: Genes significantly up-regulated in the vestibule both on the mRNA and the protein levels. Right diagram: Genes up-regulated in the cochlea on the mRNA level and in the vestibule on the protein level. Fold difference of at least 30% and FDR<0.1; (**) P<0.005 versus the other tissue.
(TIF)

Table S1 miRNA detected in cochlear and vestibular sensory epithelia.
(XLS)

Table S2 mRNA expression profile in cochlear and vestibular sensory epithelia.
(XLS)

Table S3 GO 'biological process' annotations enriched in the mRNA gene sets.
(XLS)

Table S4 Protein expression profile in cochlear and vestibular sensory epithelia.
(XLS)

Table S5 Enriched and depleted targets in the differentially expressed mRNA and protein datasets.
(XLS)

Table S6 Complete protein data.
(XLS)
