# Supplementary figures and images for: Integration of Transcriptomics, Proteomics, and MicroRNA Analyses Reveals Novel MicroRNA Regulation of Targets in the Mammalian Inner Ear
Source: PLoS One. 2011 Apr 5;6(4):e18195. doi: 10.1371/journal.pone.0018195 (PMC3071727; doi:10.1371/journal.pone.0018195)

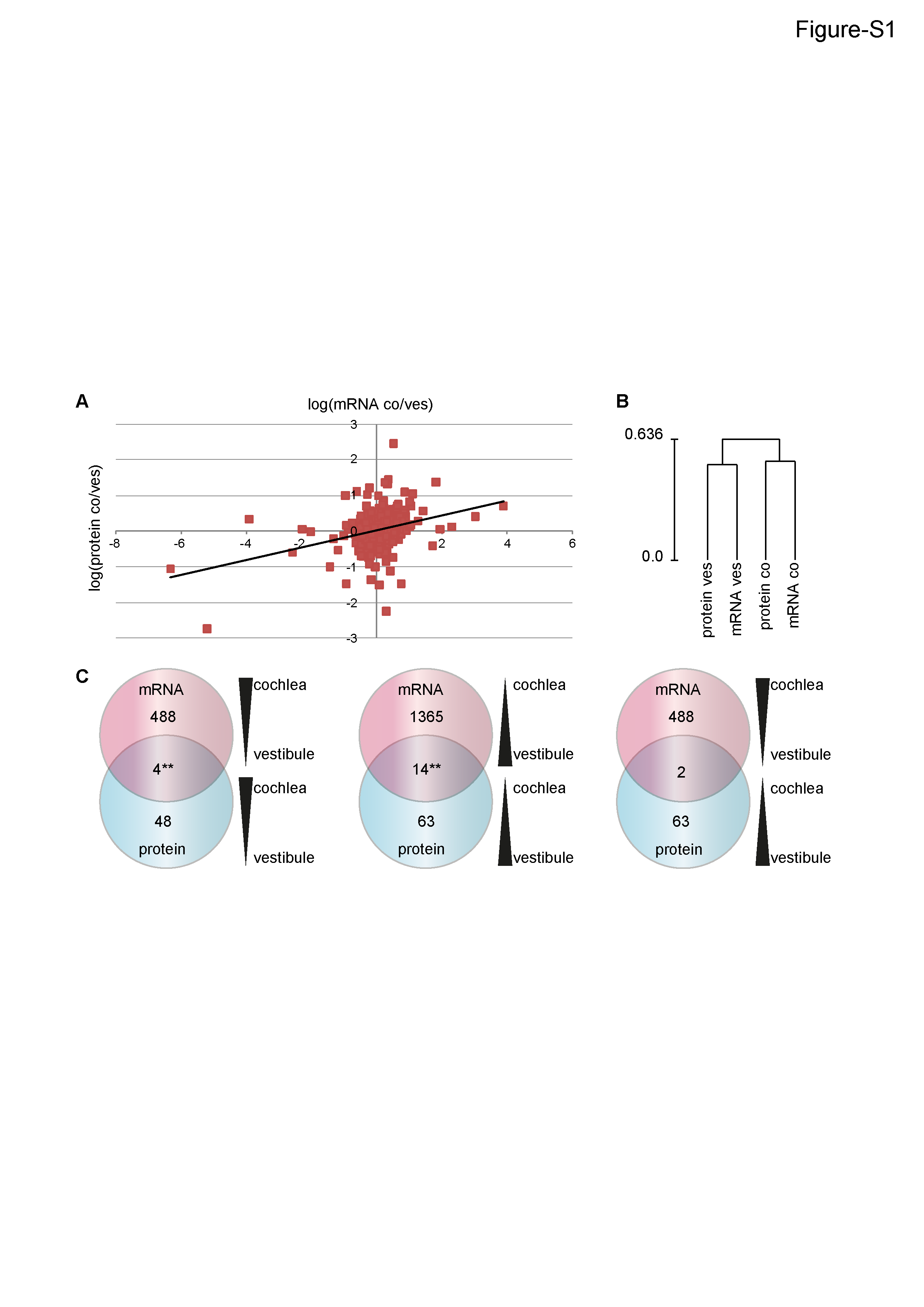

Supplement: Figure S1 — Relationship between differential cochlear and vestibular transcript and protein datasets. (A) Scatter plot representation of the protein cochlea to vestibule expression ratios obtained by the iTRAQ labeling and MuDPiT analysis and mRNA cochlea to vestibule expression ratios obtained from the Affymetrix microarray. Both expression ratios are plotted in a logarithmic scale. (B) Cluster dendrogram of the differentially expressed mRNA and protein datasets. (C) Intersection of the Affymetrix microarray (pink) and proteomic results (blue). Left diagram: Genes significantly up-regulated in the cochlea both on the mRNA and the protein levels. Middle diagram: Genes significantly up-regulated in the vestibule both on the mRNA and the protein levels. Right diagram: Genes up-regulated in the cochlea on the mRNA level and in the vestibule on the protein level. Fold difference of at least 30% and FDR<0.1; (**) P<0.005 versus the other tissue. (TIF) [file pone.0018195.s001.tif]
